# Conclusions of the digital health hub of the Transform Africa Summit (2018): strong government leadership and public-private-partnerships are key prerequisites for sustainable scale up of digital health in Africa

**DOI:** 10.1186/s12919-018-0156-3

**Published:** 2018-08-15

**Authors:** Candide Tran Ngoc, Noella Bigirimana, Derrick Muneene, Juliet Evelyn Bataringaya, Prebo Barango, Hani Eskandar, Raissah Igiribambe, Ayomide Sina-Odunsi, Jeanine Umutesi Condo, Olushayo Olu

**Affiliations:** 1 WHO Country Office, Kigali, Rwanda; 2 Rwanda Biomedical Center, Ministry of Health, Kigali, Rwanda; 3 WHO Regional Office for Africa, Brazzaville, Congo; 4 WHO Intercountry Support Team for Eastern and Southern Africa, Harare, Zimbabwe; 5 International Telecommunication Union, Geneva, Switzerland

**Keywords:** Digital health, E-health, Transform Africa summit 2018, Digital health hub, Conference proceedings, Africa

## Abstract

**Background:**

The use of digital technologies to improve access to health is gaining momentum in Africa. This is more pertinent with the increasing penetration of mobile phone technology and internet use, and calls for innovative strategies to support implementation of the health-related Sustainable Development Goals and Universal Health Coverage on the continent. However, the huge potential benefits of digital health to advance health services delivery in Africa is yet to be fully harnessed due to critical challenges such as proliferation of pilot projects, poor coordination, inadequate preparedness of the African health workforce for digital health, lack of interoperability and inadequate sustainable financing, among others. To discuss these challenges and propose the way forward for rapid, cost-effective and sustainable deployment of digital health in Africa, a Digital Health Hub was held in Kigali from 8th to 9th May 2018 under the umbrella of the Transform Africa Summit 2018.

**Methods:**

The hub was organized around five thematic areas which explored the status, leadership, innovations, sustainable financing of digital health and its deployment for prevention and control of Non-Communicable Diseases in Africa. It was attended by over 200 participants from Ministries of Health and Information and Communication Technology, Private Sector, Operators, International Organizations, Civil Society and Academia.

**Conclusions:**

The hub concluded that while digital health offers major opportunities for strengthening health systems towards the attainment of the Sustainable Development Goals including Universal Health Coverage in Africa, there is need to move from Donor-driven pilot projects to more sustainable and longer term nationally owned programmes to reap its benefits. This would require the use of people-centred approaches which are demand, rather than supply-driven in order to avoid fragmentation and wastage of health resources. Government leadership is also critical in ensuring the availability of an enabling environment including national digital health strategies, regulatory, coordination, sustainable financing mechanisms and building of the necessary partnerships for digital health.

**Recommendations:**

We call on the Smart Africa Secretariat, African Ministries in charge of health, information and communication technology and relevant stakeholders to ensure that the key recommendations of the hub are implemented.

## Background

The call for use of digital technologies to increase access to health is gaining momentum in developing countries including those of Africa [1]. This has become more pertinent with the increasing penetration of mobile technology and internet use, and calls for innovative strategies to support implementation of the health Sustainable Development Goals (SDGs) and Universal Health Coverage (UHC) on the continent [2, 3]. Digital Health (DH), which is also called eHealth, is defined as the application of Information and Communication Technology (ICT) to advance health services delivery [4, 5]. It has been successfully deployed in Africa to support several aspects of health, including management of patient and public health data, education of healthcare workers, delivery of remote health services and provision of health information and services through mobile technology [6]. Furthermore, it has also been used to monitor personal health through the use of wearable ICT devices and planning, implementation and monitoring and evaluation of health services [7].

However, the huge potential benefits of DH to advance health services delivery in Africa is yet to be fully harnessed due to critical challenges such as proliferation of pilot projects, poor coordination, inadequate preparedness of African health workforce for digital health [8], lack of interoperability of DH solutions, poor power and internet infrastructure and inadequate sustainable financing, among others [6, 9, 10]. “Pilotitis”, a word that was aptly coined by DH stakeholders, remains a major issue on the continent. It refers to the proliferation of several pilot projects, most of which have not been scaled up for wide spread community use due to the aforementioned challenges. To discuss these challenges and to propose the way forward for rapid, cost-effective and sustainable deployment of DH in Africa, health and ICT stakeholders from the continent and beyond held a meeting, the Digital Health Hub in Kigali, Rwanda from 8th to 9th May 2018 under the umbrella of the Transform Africa Summit (TAS) 2018.

TAS is an annual African Forum where global leaders and experts in ICT and development engage in constructive dialogue on the use of ICT to reduce poverty, create prosperity and increase productivity in Africa [11]. The first TAS, which was co-hosted by the Government of Rwanda and the International Telecommunication Union (ITU), was held in 2013 in Rwanda. Since then, two Summits have been held successfully in 2015 and 2017. TAS is a flagship event of Smart Africa, a bold and innovative commitment by African political leaders to accelerate sustainable socioeconomic development on the continent through the deployment of quality and affordable access to ICT, especially in underserved areas [12]. The TAS 2018, which was organized under the theme *“Accelerating Africa’s Single Digital Markets”*, hosted several parallel conferences on topical ICT issues including DH [13].

The Digital Health Hub explored ways for Africa to adopt the technology-driven innovation that is already taking place in other sectors and created a platform for health and ICT stakeholders to meet, discuss, brainstorm and network about DH in Africa and to chart a way forward for its rapid and cost-effective deployment on the continent [13]. This paper summarizes the proceedings of the Hub and provides recommendations for the scale-up of sustainable, efficient and replicable DH solutions towards achieving the health related SDGs and UHC in Africa.

## Methods

The Digital Health Hub was organized around five thematic areas which explored the status, leadership, innovations, sustainable financing of DH and its deployment for prevention and control of Non-Communicable Diseases (NCDs) in Africa. The sessions were led by moderators and panelists comprising global DH, public health and ICT professionals who engaged in expert discussions on key issues in DH through keynote presentations, experience sharing and expert opinions. The panel discussions were followed by question and answer and discussion sessions during which the audience was able to provide its contributions to the debates. The sessions ended with conclusions on the thematic areas of discussion. The hub was attended by over 200 participants from Ministries of Health (MoHs) and ICT, Private Sector ICT Operators, International Organizations, Civil Society and Academia. Three of the authors were assigned as Rapporteurs and documented the detailed discussions and conclusions of all sessions and compiled these into the Report of the hub. We extracted and reviewed the relevant sections of the Report and synthesized them into these proceedings.

## Proceedings

### Session 1: State of digital health in Africa – Landscape and countries’ roadmap

This session set the tone for discussion in the other four sessions. It focussed on understanding the progress made towards the deployment of DH in Africa, its associated challenges, and explored modalities for scaling up existing DH efforts for acceleration of health related SDGs in Africa. The keynote presentation of the session on the status of DH in Africa highlighted opportunities for DH to contribute towards strengthening health systems and for accelerating the attainment of the SDGs, including UHC. The presenter noted that mobile phone and internet penetration in the continent, which respectively stood at 80.8 and 25.1% (as against 99.7 and 47.1% at global level), has resulted in an increase in the use of DH for health services delivery. According to a 2016 WHO publication titled “Global Diffusion of eHealth”, 26 out of the 47 countries in the African Region of WHO (AFRO) have developed eHealth strategies. A regional DH strategy for scaling-up DH in Africa has also been developed [14]. In addition, the presentation noted that mHealth is the most used technology in the region (24.3% of all DH), followed by social media (21.2%), telemedicine/telehealth (20.2%), e-learning (17.2%), electronic health record (EHR) (6.7%) and big data (2.2%). The challenges impeding the successful scale-up of DH were also highlighted as including, but not limited to: non-interoperability among various DH systems; proliferation and duplication of DH solutions and projects; weak capacity for monitoring and evaluation; high cost of scaling-up; poor coordination and limited impact on the health system and the non-use of health system-based approaches for deployment of DH projects.

A new initiative, which is the result of a partnership between the African Regional Office of WHO (AFRO) and ITU was presented. This project, which aims at fast tracking the deployment of DH technologies in Africa, has four main components, namely: 1) addressing the problem of interoperability; 2) capacity building in DH; 3) strengthening partnerships for DH and 4) promoting connectivity of medical devices and equipment to DH.

In the panel discussion that followed the keynote presentation, it was stressed that DH cannot exist in isolation but within the framework of a strong health system and that the development of large scale and cost-effective business models are required for effective scale up of DH. Furthermore, the importance of south-south cooperation mechanisms in rapidly scaling up DH in the Region [15] and the use of a digital health atlas and index to measure progress in the maturity of DH implementation were also highlighted. During the question and answer session, the participants listed capacity building on DH technologies, documentation of achievements including economic studies to build an investment case for DH and the involvement of the private ICT companies in supporting DH as urgent imperatives. The African Alliance of Digital Health Networks, a body which aims to develop digital health leaders and entrepreneurs in the Region was launched at the end of this session.

The session concluded that good progress has been made in the deployment of digital technologies for health service delivery in Africa although many challenges persist. DH cannot function in isolation and be should be integrated into existing health systems, Furthermore strong political commitment and south-south cooperation are critical for scaling up DH in Africa.

### Session 2: Government leadership in digital health, and the need to break silos and mitigating “pilotitis”

Strong leadership and good governance is critical to achieving good public health outcomes. Against this backdrop and conclusions of session 1, session 2 focussed on the importance of governance in scaling up DH in Africa. The key findings of a global survey on DH leadership [16] were presented to set the tone for discussion. The survey findings showed that DH can significantly contribute to expansion of health coverage, empower patients and communities in management of their own health as well as improve quality of care by centralizing expertise and connecting community health workers with specialists. DH could also reduce costs and increase the efficiency of health care delivery. The survey results also noted that the fast growing penetration of mobile technology could contribute globally to huge expansions in DH by 2030. Although 69 and 76 out of 116 countries surveyed respectively had eHealth strategies and electronic health information systems; however slow or lack of implementation remains a challenge. The survey also showed that, while the numbers of DH projects in developing countries increased by more than 30% between 2005 and 2011; most of them were fragmented and two thirds were still at informal pilot stages. The survey therefore proposed three DH leadership mechanisms namely 1) An MoH-led mechanism in collaboration with other relevant Ministries; 2) A Government-wide DH technology Agency mechanism in which MoH drives DH but is a client to the government-wide Agency, and 3) A dedicated DH Agency Mechanism.

Insufficient and inadequate capacity for DH governance and leadership were also mentioned as critical challenges in Africa; and the fundamental roles of Governments in addressing the aforementioned challenges could thus not be overemphasized. These include the need for all Governments to develop and implement DH strategies [17] and to provide strong leadership to monitor and coordinate the often fragmented DH ecosystem. Several country and regional experiences of how strong leadership and good governance has driven expansion of DH were presented. In the Republic of Benin, this resulted in the development and effective implementation of a national eHealth strategy while in Abia State of Nigeria, the implementation of an eHealth strategy resulted in increased access to health care, reduced cost of producing health services and improved quality of health consultations. However, issues such as internet connectivity and cyber insecurity were highlighted as critical challenges. At the regional level, the success of the Regional East African Community Digital Health Initiative (Digital REACH), a project of the East African Community (EAC) in expanding DH solutions in EAC countries, was also presented. The question and answer session highlighted the need to address the problem of interoperability of health data across national borders and the need for cyber security regulations to ensure data protection and security.

The session concluded and recommended that effective government leadership [18], coordination and context specific DH solutions are needed in Africa. Government leadership should focus on creating enabling environments including national digital health strategies, regulatory, coordination and sustainable financing mechanisms and building necessary partnerships to ensure scalable, replicable and cost-effective DH initiatives which are well adapted to the local African context. In addition, the establishment of common national DH platforms, nationally owned and led, are also required to successfully scale up the implementation of DH.

### Session 3: Innovation for digital health – Biotech, drones, big data, artificial intelligence: Where are the quick wins?

This session discussed the current state of innovations and pragmatic deployment of DH to improve health outcomes and expand access to health services in Africa. Several innovative interventions in the areas of health data management, training and implementation of primary health care services were presented.

In Rwanda, Babyl, a digital health care provider, has developed and successfully used a virtual medical consultation platform to reduce the time it takes to consult a medical doctor. This system ensures that valuable time is not wasted waiting in long queues to be attended by health practitioners. Zenysis Technologies have developed a system that integrates health data from multiple sources such as surveys and routine information systems, allowing easy comparison, as well as forecasting of health events. This consequently increases the efficiency of the overall health system. The Carnegie Mellon University is using innovative approaches to train African students at graduate and postgraduate levels in the areas of data science, cyber security and software engineering to specifically address the technological needs of the continent and could contribute to accelerating the deployment of DH. In Ondo State of Nigeria, the Primary Health Care Agency successfully used DH to improve the quality of health data and information; to remotely train healthcare workers using video teleconferencing facilities and to disseminate health information through the use of SMS. The Global Alliance for Vaccines and Immunization (GAVI) is using an innovative system called INFUSE (Innovation for Uptake, Scale up and Equity in Immunisation) to leverage digital technologies to close the gaps in immunization globally. GAVI is also supporting an innovative project which uses drones to supply lifesaving blood supplies to remote health facilities in Rwanda.

The panellists however noted that scaling up these innovations requires an enabling environment such as legislation, appropriate technology, uninterrupted power supply and a single platform to avoid duplication and wastage of resources. The panellists further mentioned that TAS plays an important role in bringing stakeholders together to address these challenges and recommended to invite a broader range of stakeholders to future Summits. Participants at this session provided suggestions on how to create an enabling environment for scaling up these innovations on the continent during the question and answer session. They stressed the need for capacity building, leadership and the importance of data security as some of the prerequisites for scaling up DH innovations. Data security could be assured through the use of aggregated data (as opposed to individual data), strong data encryption, anonymization and restriction of access to data for certain categories of users. Furthermore, it was suggested that the development of innovations should be based on clearly identified local public health problems to which they can be applied [19]. The session participants also opined that the continent is not ready for the replacement of health practitioners with artificial intelligence. Rather, the DH expertise of the concerned practitioners should be enhanced to improve their efficiency.

The session concluded that people should be at the core of DH innovations in Africa, and that such innovations should be based on clearly identified and very specific public health needs. The session also concluded that building the capacity of health workers for DH is critical.

### Session 4: Universal health coverage and NCDs – Challenges and digital health solution

This session discussed the role of DH in the prevention and control of NCDs along with the bottlenecks related to the implementation of DH. NCDs are emerging as a major public health challenge in Africa and a significant proportion of the premature mortality from NCDs occurs on the continent. Being chronic diseases, they require long-term access to cost-effective interventions across the spectrum of prevention, early diagnosis, treatment and palliative care. The behavioural risk factors for NCDs; tobacco use, unhealthy diets, physical inactivity and harmful use of alcohol are also increasing in Africa. The implementation of best buys such as tobacco and alcohol control interventions, increasing physical activity, vaccination against human papilloma and hepatitis B virus and screening for cervical cancer is indispensable to reverse the growing trend of NCDs in the region.

The management of NCDs on the continent is fraught with several challenges such as barriers to regular medical consultations and diagnostic tests, inadequate access to health promotion messages, essential medicines and poor adherence to medications all of which could be addressed by DH solutions. The high mobile technology and internet penetration in the continent provides opportunities to leverage DH for the prevention and control of NCDs. This was demonstrated by a few examples such as the global Be He@lthy Be Mobile [20], a WHO/ITU collaborative initiative which assists governments in using mobile technology to reinforce their existing national health activities to prevent, manage, and treat NCDs and their risk factors. Examples of this initiative include the mDiabetes project in Senegal [21] and the mCervical Cancer initiative in Zambia [22]. AMREF, an African Non-Governmental Organization also uses mobile technology to train first-line health workers and to educate communities in NCDs prevention, including screening and referral.

This session concluded that DH could play an important role in a patient-centric model to achieve the universal coverage of NCDs prevention and control interventions in Africa. These roles range from providing reminders to patients to take their medications and to live healthily, to assisting them in obtaining medical consultations and their medications. The session also concluded that innovative strategies to finance the deployment of DH for NCDs, such as allocating revenues generated from increasing taxation on tobacco products, alcoholic and sugar-sweetened beverages should be explored. Furthermore, task sharing and shifting supported by DH was also proposed as a feasible option for using DH in NCDs prevention and management.

### Session 5: Investing in digital health – Business models and public-private-partnerships

The last session discussed the business models and role of Public-Private Partnerships (PPP) in scaling up DH investments in Africa. Sustainable investments such as infrastructure, human resource and finances are needed to expand DH in Africa. However, according to WHO global survey on eHealth, the spread of financial investment for DH in the Africa region varies among countries; with 72% of the funding being provided by external Donors. This situation raises the critical issue of sustainability. This session therefore examined different models designed to ensure sustainable financing of DH on the continent which could be viewed both from the perspective of increasing funding as well as saving costs.

The critical roles of the African private sector including Development Banks in increasing the financing of DH in Africa was highlighted, with the necessity for African governments to engage with the sector to develop PPPs [23]. In this regard, African governments were encouraged to collectively negotiate the prices of DH through their regional economic communities which could increase their bargaining power and reduce the cost of DH on the continent. An American NGO (PATH) brokered DH project in Zambia, which comprises a mix of interoperable DH solutions, was proposed as a model for successful deployment of successful PPPs in Africa. This project significantly reduced the malaria incidence in the Southern Region of Zambia from 8 to 0.6%. Also, it was emphasized that open source DH applications should be seen as public health goods to address the digital divide in Africa. The case of the District Health Information System (DHIS-2), a free and open-source software for health data management was used to buttress this point. Importantly, the return on DH investments should not be seen only from the financial perspective but also in terms of lives saved, better health, and impact on strong health systems. There is therefore crucial need for further research to elicit the impact of DH in these areas [24].

In the question and answer session, the participants called for more ownership and commitment of African governments to DH as a means to increase sustainable financing and avert failure of projects. It was also emphasized that, while pilot projects are useful to test ideas and produce evidence before scaling up; they should be demand-driven. It is also important to understand the cost of technology solutions, customize DH solutions and adapt the technology to the context. The session concluded that African governments should play critical roles in ensuring sustainable financing of DH [25]. In this regard, an investment case and business model should be developed [19]. In addition, the participants at the session concluded that there is need to document achievements and lessons learnt in the implementation of DH projects in Africa to demonstrate return on investments so as to attract private investors [1, 19].

## Conclusions

The digital health hub was a good platform to deliberate and conclude on key issues about rapid, sustainable and cost-effective expansion of DH solutions to strengthen health service delivery in Africa. Many critical insights for scaling up DH in Africa and other similar settings originated from the hub. First, while DH offers a major opportunity for strengthening health systems towards the attainment of the health related SDG goals (including UHC) on the continent, there is need to move from Donor-driven pilot projects to more sustainable and longer-term nationally owned program to reap its benefits. Second, strong health systems and UHC should be at the very core of every DH initiative in Africa. Third, the importance of government ownership, leadership, good governance and PPPs in sustainable financing and successful scale up of DH in Africa cannot be overemphasized. Fourth, DH projects should be people-centred so as to support communities especially the hard-to-reach ones to access equitable and good quality health services. Fifth, DH initiatives should be demand-driven rather than supply-driven in order to avoid fragmentation and to ensure that they address specific public health problems. Sixth, the scaling up of DH on the continent requires well trained health workers who are versed in DH and health services delivery thus capacity building is an imperative on the continent. Seventh, the security of public health data is a cross-cutting issue which needs to be comprehensively addressed in Africa.

## Recommendations

We call on the Smart Africa Secretariat, Ministries in charge of health and ICT of all African countries and all relevant stakeholders to ensure that the key recommendations of this meeting are implemented. Specifically we call on African Governments to provide the required leadership for DH on the continent. We also call on the private sector especially the private ICT Operators to support African governments to establish PPPs for the scale up of DH solutions on the continent. Lastly, we call on African academic institutions to strengthen training on DH for health and ICT workers.

## Abbreviations

AFRO: WHO Regional Office for Africa
DH: Digital Health
EAC: East Africa Community
EHR: Electronic Health Record
GAVI: Global Alliance for Vaccines and Immunization
ICT: Information and Communication Technology
ITU: International Telecommunications Union
MoH: Ministry of Health
NCDs: Non-communicable Diseases
PPP: Private-Public Partnerships
SDGs: Sustainable Development Goals
TAS: Transform Africa Summit
UHC: Universal Health Coverage
